# Advances in Metal–Organic Frameworks for the Removal of Chemical Warfare Agents: Insights into Hydrolysis and Oxidation Reaction Mechanisms

**DOI:** 10.3390/nano13152178

**Published:** 2023-07-26

**Authors:** Madeleine C. Oliver, Liangliang Huang

**Affiliations:** School of Sustainable Chemical, Biological, and Materials Engineering, University of Oklahoma, Norman, OK 73019, USA; madeleine.oliver@ou.edu

**Keywords:** metal-organic framework, Zr-MOFs, chemical warfare agent, degradation, hydrolysis, oxidation, reaction mechanism, computational research

## Abstract

The destruction of chemical warfare agents (CWAs) is a crucial area of research due to the ongoing evolution of toxic chemicals. Metal–organic frameworks (MOFs), a class of porous crystalline solids, have emerged as promising materials for this purpose. Their remarkable porosity and large surface areas enable superior adsorption, reactivity, and catalytic abilities, making them ideal for capturing and decomposing target species. Moreover, the tunable networks of MOFs allow customization of their chemical functionalities, making them practicable in personal protective equipment and adjustable to dynamic environments. This review paper focuses on experimental and computational studies investigating the removal of CWAs by MOFs, specifically emphasizing the removal of nerve agents (GB, GD, and VX) via hydrolysis and sulfur mustard (HD) via selective photooxidation. Among the different MOFs, zirconium-based MOFs exhibit extraordinary structural stability and reusability, rendering them the most promising materials for the hydrolytic and photooxidative degradation of CWAs. Accordingly, this work primarily concentrates on exploring the intrinsic catalytic reaction mechanisms in Zr-MOFs through first-principles approximations, as well as the design of efficient degradation strategies in the aqueous and solid phases through the establishment of Zr-MOF structure–property relationships. Recent progress in the tuning and functionalization of MOFs is also examined, aiming to enhance practical CWA removal under realistic battlefield conditions. By providing a comprehensive overview of experimental findings and computational insights, this review paper contributes to the advancement of MOF-based strategies for the destruction of CWAs and highlights the potential of these materials to address the challenges associated with chemical warfare.

## 1. Introduction

Chemical warfare agents (CWAs) are lethal weapons of mass destruction that have been utilized in military conflict since their introduction in World War I. The first ever recorded large-scale chemical attack was the release of chlorine gas by German troops against the Allies in 1915, which resulted in more than a thousand casualties [1,2]. Once this proof of their extreme toxicity and devastating effects came to light, continuous efforts were made to investigate and store various toxic compounds for use as fatal devices against soldiers and civilians [1,3]. The most significant use is dated to the Iraq–Iran War, where a massive chemical attack on the city of Halabja resulted in 200 fatalities in 1988 [4]. While the production and use of CWAs were prohibited by the Chemical Weapons Convention shortly after, in 1993 [2], their evolution remains ongoing, and military personnel face growing uncertainty in complex battlefield environments. Terrorist organizations have also increased the relevancy of chemical warfare agents used against civilians, the most notable incidents being fatal nerve agent attacks in the city of Matsumoto and a Tokyo subway system in the late 1990s. Advancements in personal protective equipment that incorporate highly efficient filtration media for the rapid capture and decomposition of CWAs are urgently needed to mitigate the extreme threat these chemical weapons pose to individuals and military operations.

Measures to protect against CWAs must occur before the chemicals reach their biological targets, as the time frame to apply effective treatment after exposure can be as short as minutes [3]. Current protective technologies have been developed using solid materials such as activated carbons, which can collect and retain CWAs released into the atmosphere but cannot decompose them [5]. Metal oxides, mesoporous silica, zeolites, and surfactants have also been investigated [6,7]; however, complications including low adsorption capacities, competition with atmospheric constituents, deactivation of active sites, and slow reaction kinetics render these materials generally incapable of adequate chemical decomposition [7,8,9]. Research efforts have thus shifted to the design of adsorbents, particularly nanoporous materials, with the ability to both capture and efficiently degrade CWAs under operationally relevant field conditions [10]. Metal–organic frameworks (MOFs) are a class of porous crystalline solids that have sparked interest in this area. MOFs consist of metal ion clusters connected by multidirectional carbon-based bridging linkers, yielding pores with sites for both organic and inorganic chemistry [11]. With tunable networks that provide customizable chemical functionalities, MOFs are adjustable to dynamic environments, making them useful in a wide variety of practical applications, such as gas storage, separations, drug delivery, chemical sensing, and catalysis [11,12,13]. In recent years, MOFs have been identified as superior materials for detecting and breaking down target species, owing to their excellent adsorption, reactivity, and catalytic abilities [1]. Coupled with their exceptional porosities and large surface areas, many MOFs provide an ideal setting for the selective capture and detoxification of CWAs [1,8,9,11,14,15,16,17].

Although progress has been made in using MOF-based materials as catalysts for CWA degradation [16], it remains unclear what combination of features enables efficient chemical breakdown in the solid phase and under realistic environmental conditions of humidity. Furthermore, many puzzles still exist regarding how those features may change with respect to the specific toxic chemical and the mechanism of detoxification. Understanding the interactions of various MOF structures with various CWAs in the presence of varying levels of atmospheric moisture is essential to establishing the design rules for a single MOF to achieve universal toxic chemical degradation in dynamic environments. This mini-review discusses advancements in the computational and experimental studies of MOFs for the degradation of nerve agents, blister agents, and their simulants, with a primary focus on degradation via hydrolysis and oxidation strategies in zirconium-based MOFs. Key design considerations for efficient degradation concerning CWA type and reaction mechanism are summarized, and insight is given into the future research directions necessary for filling the gaps in current understandings.

## 2. Properties of Chemical Warfare Agents

### 2.1. Nerve Agents, Vesicants, and Their Simulants

CWAs can be divided into several types, including nerve agents, blister agents (vesicants), blood agents, tear agents, and choking agents. The most common are nerve agents and vesicants, which can be further split into categories based on their chemical structures. Nerve agents belong to the chemical group of organophosphorus compounds. They can be either G-type or V-type, where G-type indicates fluorine (GB, GD, GF)- or cyanide (GA)-containing compounds, and V-type (VX) indicates sulfur-containing compounds [18]. G-type nerve agents, such as sarin (GB) and soman (GD), are among the most toxic of CWAs, causing inhibition of proper muscle responses in the body within seconds of exposure and death within minutes [18,19]. Blister agents are less threatening than nerve agents, primarily intended to injure rather than kill people [18]. There are three categories of blister agents, including mustards (HD, HN-1, HN-2, HN-3), arsenicals (L, HL, PD), and urticants (CX). Mustards are the most prominent members of the vesicant family [20], existing in the form of nitrogen mustard (HN) or sulfur mustard (HD). Damage from these CWAs usually occurs in the tissues, in the form of blisters on the skin or irritation of the eyes [21]. Still, like nerve agents, vesicants are typically disseminated as vapors or liquids and can be inhaled and readily absorbed through the skin. While nerve agents range in persistency, all vesicants are relatively persistent, making fatality a possibility depending on exposure conditions (especially in the case of repeated exposures).

**Figure 1:**
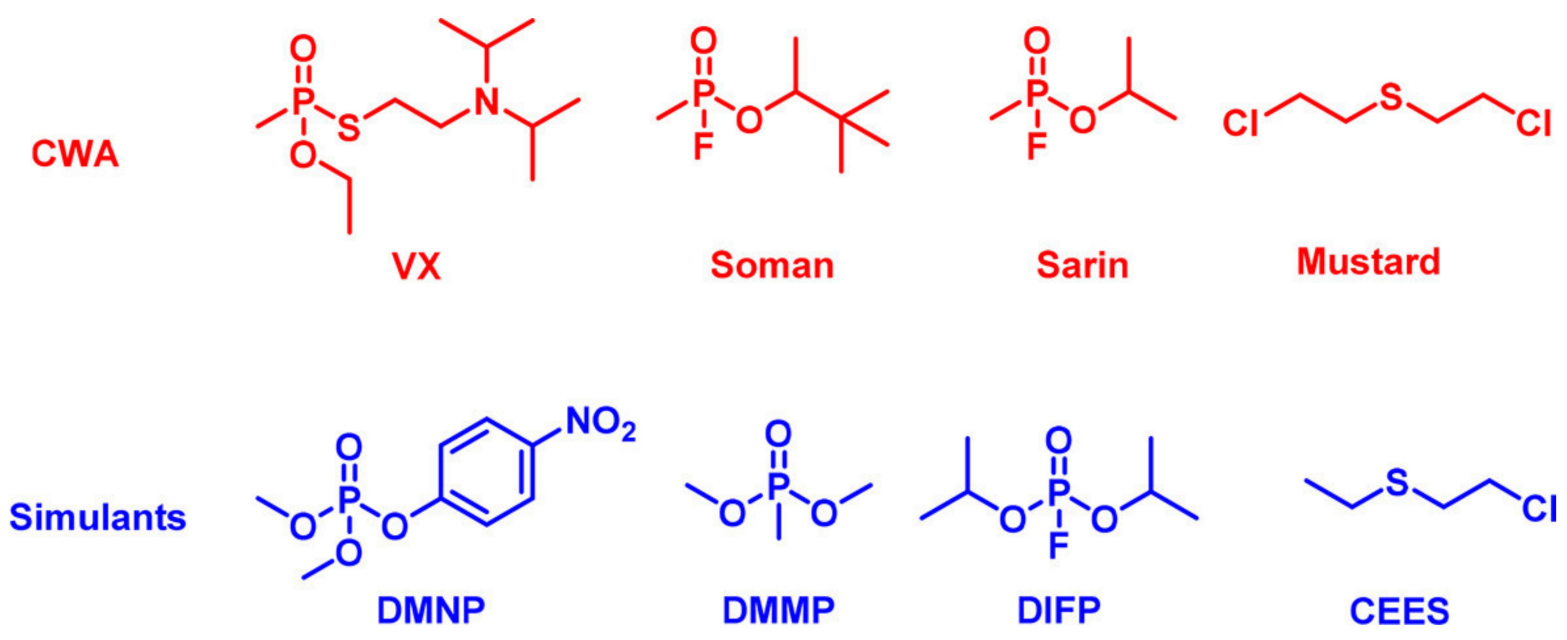
Representative examples of CWAs discussed in this work and their commonly used, less toxic simulants [16]. Reprinted with permission from ref. [16]. Copyright 2020 American Chemical Society.

The literature reviewed in this work is primarily concentrated on the degradation of the nerve agents GB, GD, and VX and the blister agent HD. Due to their high toxicity, experimental research using CWAs can be hazardous and is restricted in most laboratories. The most straightforward approach to overcoming this challenge is to detect and analyze respective CWA simulants, as they mimic the chemical behavior of CWAs by exhibiting similar chemical and physical properties with lower toxicity [1]. The chemical structures of GB, GD, VX, and HD are shown in Figure 1, along with the chemical structures of their commonly used simulants.

We note that while these simulants have been widely utilized in experiments to help predict and correlate the degradation mechanisms and behaviors of their corresponding CWAs [1], it is unrealistic to expect that any simulant can satisfactorily represent all the properties of a given CWA [22]. For example, studies of organophosphate agent and simulant interactions in aqueous solution have reported that DMMP and DIFP mimic the interactions of soman and sarin with water reasonably well [23,24]. In contrast, studies on the adsorption of nerve agents in MOFs have found that the adsorption properties of soman and sarin are poorly correlated with those of DMMP and DIFP, respectively [25,26]. DFT models of the hydrolysis reaction mechanism have also revealed that many commonly used simulants in the literature, like DMMP, demonstrate large energy barrier deviations from GB and GD [27], which underlines the risk of choosing simulants based on the literature precedent alone. In addition to closely matching the chemical structure of the CWA, the selection of an appropriate simulant evidently requires detailed knowledge and evaluation of the properties that strongly affect the process at hand, as the simulant that is most appropriate to simulate a particular process in a particular environment may not be the best choice for all processes in all environments [22,28]. First-principles calculations and other computational approaches have thus become of special importance in this field, both for gaining insight into the relevant properties of ideal simulants for experimental study and for modeling the behaviors of real CWAs in a given system of interest.

### 2.2. Degradation Mechanisms

**Scheme 1:**
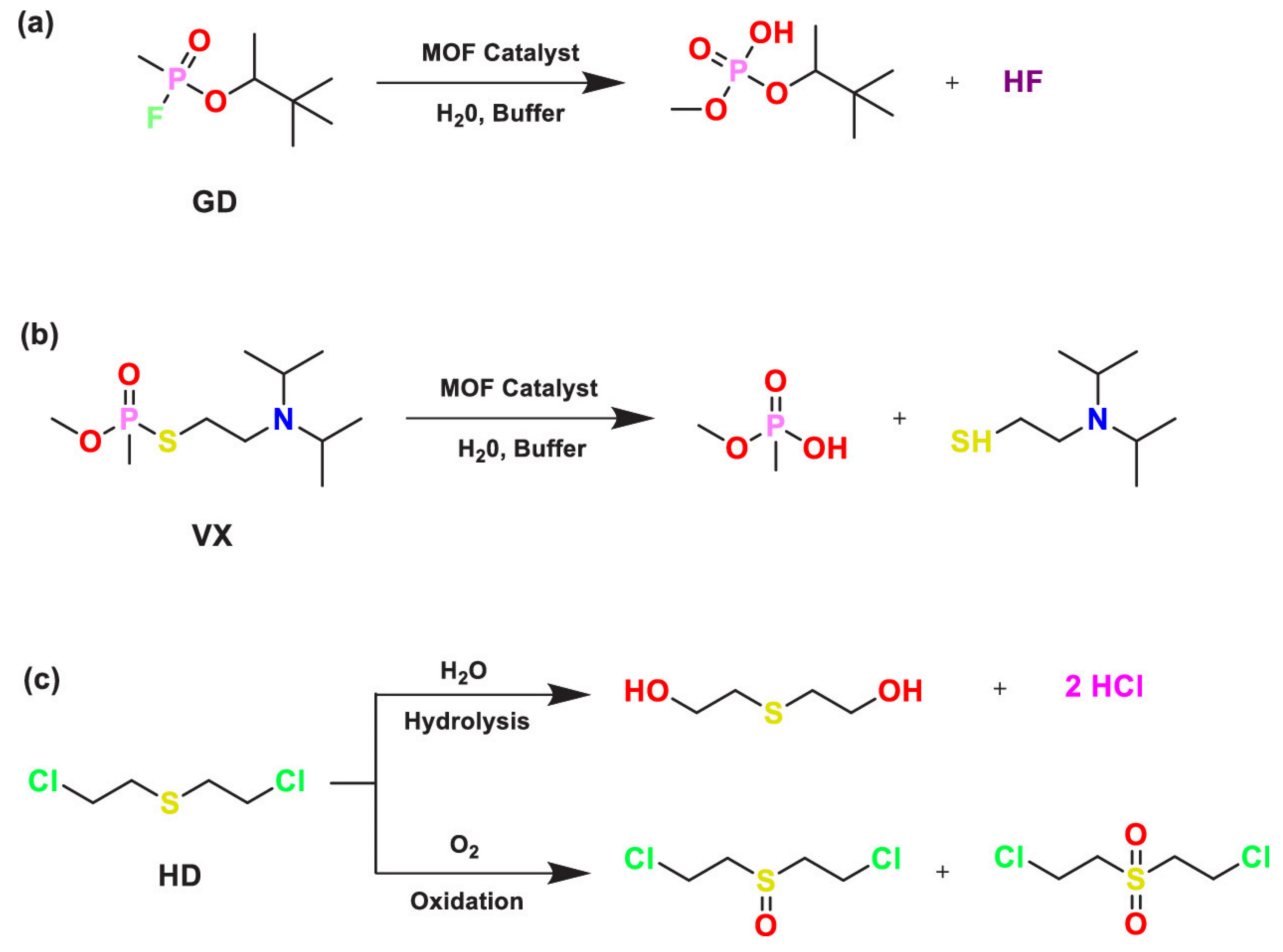
Detoxification/hydrolysis pathway of CWAs. (**a**) Nerve agent GD; (**b**) nerve agent VX; (**c**) vesicant agent HD [1,6,17]. Reprinted with permission from ref. [1] and reproduced with permission from refs. [6,17]. Copyright 2021 American Chemical Society, 2017 Elsevier, and 2016 Wiley-VCH, respectively.

The proposed mechanisms of CWA degradation in the current literature are predominantly hydrolysis and oxidation [1]. Nerve agent removal occurs mainly via hydrolysis but differs depending on the type of CWA, particularly with respect to the reaction products. As shown in Scheme 1, hydrolysis of the nerve agent GD generates pinacolyl methylphosphonic acid (PMPA) and hydrofluoric acid. In contrast, ethyl methylphosphonic acid (EMPA) and the organosulfur compound 2-(diisopropyl)aminoethanethiol (DIAT) are the hydrolysis products of VX [29]. GB, not pictured here, hydrolyzes similarly to GD, with a hydrofluoric acid product accompanied by isopropyl methylphosphonic acid (iPMPA). Besides differences in their products, GB and GD have greater volatility and reactivity to water than VX, persist for a shorter time in the environment (hydrolyze at a faster rate), and have less complex reaction mechanisms [30]. In general, the various alkyl methylphosphonic acid (AMPA) compounds produced in the degradation of these and other nerve agents can all be even further hydrolyzed into the stable product of methyl phosphonic acid (MPA).

Degradation strategies for HD include hydrolysis, dehydrohalogenation, and selective oxidation [11,31,32]. An important obstacle of degradation via hydrolysis, which arises in both mustard agent and nerve agent removal, is the formation of acid byproducts that can lead to catalyst poisoning and inhibition of subsequent reactions. While this issue can often be confronted with modifications to materials and operating conditions, the hydrolysis of HD (Scheme 1c, top) is also primordially rate-limited by the compound’s immiscibility in water [33,34]. Likewise, the degradation of sulfur mustard by dehydrohalogenation is considered too slow [11] since it typically requires a high pH environment that is corrosive to most materials [34]. Given these roadblocks, oxidation is the most effective mechanism for HD removal in real-time applications. Sulfur mustard oxidation can be partial or complete, producing desirable and undesirable products. Partial oxidation to sulfoxide (HDO; Scheme 1c, bottom left) is an attractive decontamination strategy, as this product displays improved chemical stability that makes it rather inert towards biological systems [33]. On the other hand, complete oxidation produces the di-oxidized product sulfone (HDO_2_; Scheme 1c, bottom right), which has vesicant properties similar to the parent HD [35]. Selective partial oxidation is therefore required for the safe degradation of HD to the nontoxic HDO [33,35]. For a 100% selective reaction to be achieved, mild oxidizing agents such as photosensitizers must be used [31] and exceedingly careful monitoring must be enforced to avoid detrimental over-oxidation to HDO_2_ [33].

## 3. CWA Removal by Metal–Organic Frameworks (MOFs)

### 3.1. Structural Features of Promising MOFs

While MOFs offer a large variety of structures for use against CWAs and emerging hazards, their exploitation depends critically on understanding the structure–activity relationship needed for efficient uptake and decomposition under operationally relevant battlefield conditions. A principal quality to consider when evaluating these materials for CWA removal is thus their stability and reactivity toward environmental constituents, particularly water. MOF functionality in humid conditions can be determined by several thermodynamic and kinetic factors, including the strength and geometry of metal–linker coordination bonds, pore sizes and connectivity, hydrophilic or hydrophobic framework components, and metal ion valency [36]. Many MOFs show limitations of weak mechanical and chemical stability in the presence of water that stems from hydrophilic functional groups, easily accessible active sites, water-susceptible linkages between metal nodes and ligands [37], or some combination of the three. In chemical reaction applications, these features can also lead to problems beyond structural stability, such as hindered target species adsorption and slow reaction kinetics. This section outlines the key attributes contributing to strong water stability and performance in MOFs and introduces the types of MOFs considered well suited for CWA degradation via hydrolysis and oxidation.

#### 3.1.1. Nodes and Linkers

The metal ion may be the most important factor when evaluating MOF stability in humid conditions. A study by Lee et al. [38] showed that the chemical stability of MOFs is inversely correlated to the strength of the coordination bonds between metal centers and their ligands [39]. MOFs with weak linkages are susceptible to linker hydrolysis or linker exchange, where water attack on the nodes leads to the breaking of these bonds, resulting in phase changes or framework collapse. One method for combatting thermodynamic favorability towards framework degradation is designing MOFs using Pearson’s HSAB theory [40], with hard–hard or soft–soft node–linker combinations. MOFs with high oxidation state metals (such as Cr^3+^, Fe^3+^, Al^3+^, and Zr^4+^) and organic carboxylate ligands have been widely investigated for applications involving humidity [37,41,42], as metals with small ionic radii and high positive charges make hard acids, thus binding very strongly to the oxygen atoms of carboxylate ligands, which are hard bases. Some examples of highly thermodynamically stable MOFs include MILs, ZIFs, pyrazolate-containing frameworks, and zirconium-based MOFs [43]. Zr-based MOFs, specifically, have been proven to possess unprecedented stability in humid environments [13,41,44], owing to the Coulombic interactions between their negatively charged termini of linkers and highly oxophilic Zr^IV^ centers [43]. MOFs with heavier low-valent metals coordinated to soft ligand donors have also been considered [45] due to the likelihood of stronger host–guest binding interactions from metal centers with radially expanded valence orbitals. However, it is understood that softer metal–ligand bonds tend to be more rapidly hydrolyzed than their hard–hard counterparts [45].

From a kinetic standpoint, framework degradation can be combatted by incorporating chemically unreactive metal atoms. A study by Kang et al. [46] on the isotypic MOFs MIL-53-Al, MIL-53-Cr, and MIL-47-V showed that chemical stability in water decreases in the order of Cr > Al > V or with respect to the degree of metal ion inactivity [36]. Likewise, a study by Towsif Abtab et al. [47] described a Cr^III^-based MOF with extraordinary water stability enabled by the metal’s inertness. Nevertheless, the unreactive nature of metal ions such as Cr^III^ makes synthesizing crystalline MOFs of this type extremely difficult [48,49].

#### 3.1.2. Pore Sizes and Connectivity

In the absence of thermodynamic stability, one way of avoiding MOF hydrolysis in humid conditions is through steric factors, like increasing the coordination of the metal nodes [50]. Higher node connectivity can effectively block the access of water molecules to vulnerable metal–linker bonds, eliminating the possibility of framework collapse. For example, a study by Emerson et al. [51] demonstrated that an unconventional triaminepentacarboxylate acid ligand forms a highly linked porous coordination polymer with Cd^II^ metal ions, whose exceptional connectivity yields a hydrolytically stable MOF towards CO_2_ sorption. However, high connectivity in MOFs is often accompanied by small pore openings, which limit the accessibility and abundance of potential binding sites to target species in chemical reaction applications [7]. In fact, a key issue with many MOFs for toxic species removal (with or without chemical stability) is pore openings that are smaller than or comparable to the molecular sizes of CWAs.

In chemically stable MOFs, a well-known strategy for improving site accessibility is defect engineering, or the removal of organic linkers to increase the aperture size of the framework and decrease the coordination of the metal nodes [17]. Defect engineering allows for the creation of hierarchical porosity in MOFs, which can excavate hidden active sites and increase the volumetric uptake of toxic chemicals [52,53]. Although promising, defect characterization and topological tuning to obtain a precise balance between site access and desired kinetic efficiency is a major unsolved challenge [54]. While linker removal can increase pore volume, enabling better adsorption and diffusion of guest species, it can also result in the formation and exposure of undercoordinated sites, which interact strongly with guest molecules and decrease kinetics. An example of this was shown in a defect study by Wang et al. [55], who observed that the introduction of missing linkers to the UiO-66 Zr-MOF promoted interactions between adsorbed IPA and coordinatively unsaturated metal sites that were strong enough to outweigh the effects of increased pore volume, ultimately decreasing adsorbate diffusion through the framework. Another defect study by Ghosh et al. [56] additionally revealed that structural impurities in the form of missing linkers in UiO-66 made the MOF more hydrophilic. These combined results suggest that in CWA degradation operations, defect exposure would eliminate one problem while introducing another; CWA removal would be less hindered by small pore openings and poor site access but more hindered by reaction inhibition from the strong binding of decomposed products [19,57] or competition with environmental water for active sites [58]. Given these drawbacks, MOFs with intrinsic hierarchical porosity present as favorable alternatives.

#### 3.1.3. Hydrophilicity and Hydrophobicity

**Figure 2:**
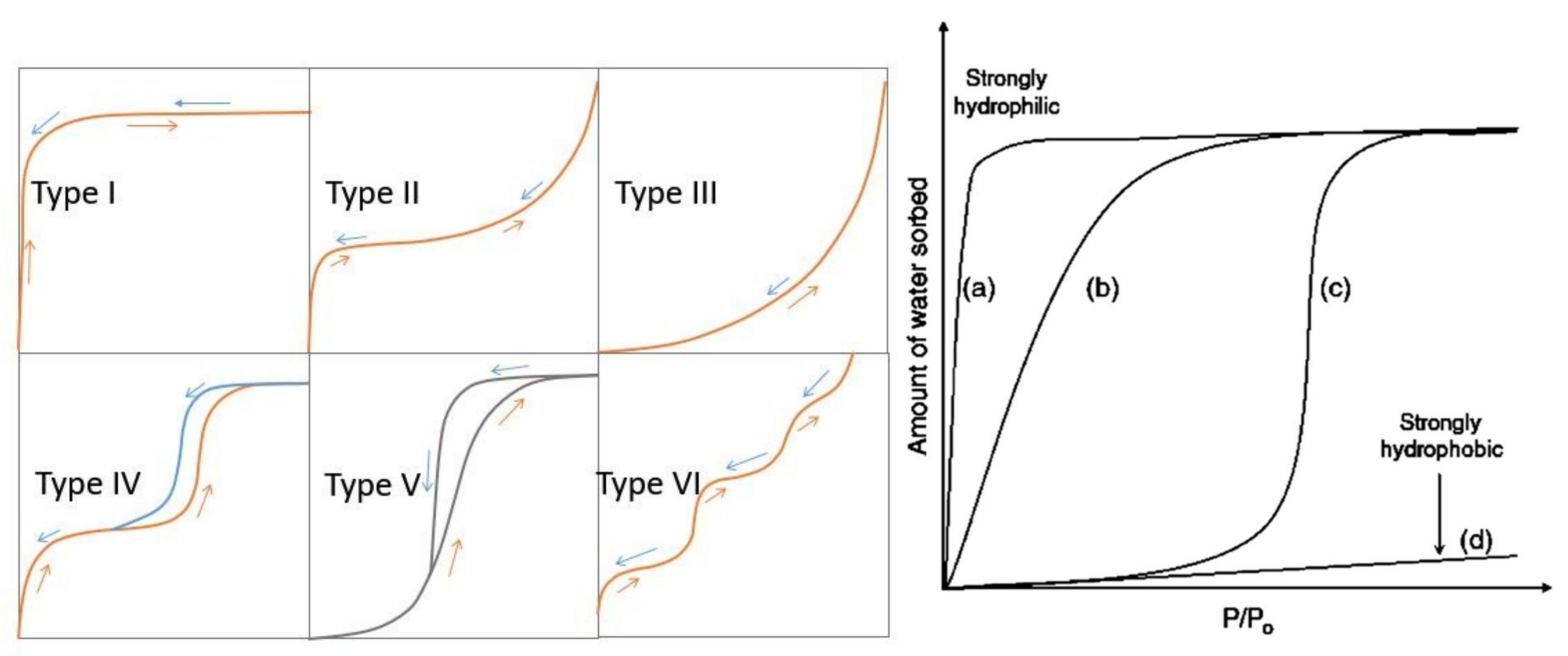
Adsorption isotherms for water in different types of nanoporous adsorbents near room temperature. IUPAC classification of adsorption isotherm types (**left**) and adsorption patterns with respect to hydrophilicity (**right**) [50,59,60,61]. Reprinted with permission from ref. [60] (**left**) and from refs. [50,59] (**right**). Copyright 2019 Royal Society of Chemistry, 2014 American Chemical Society, and 2008 Elsevier, respectively.

In any case, the overall effectiveness of an MOF towards toxic species removal depends heavily on its affinity for water. While strong node–linker bonds or high activation energies can prevent issues like framework hydrolysis, extreme hydrophilicity or hydrophobicity of the framework can seriously hinder or interfere with the efficiency of the degradation reaction. The shape of the water vapor adsorption isotherm can indicate the level of hydrophilicity or hydrophobicity in a nanoporous adsorbent material, as shown in Figure 2. Adsorbents with type I, type II, and type IV isotherms are classified as hydrophilic. In MOFs of this type, environmental water will adsorb into the pores at very low pressures, dispersing to available active sites to participate in adsorbate–adsorbent interactions. The more hydrophilic the material, the more water–framework interactions will precede interactions with target species, lowering active site accessibilities and target species reaction rates. These obstacles make hydrophilic MOFs a poor choice for chemical reaction applications in settings with large atmospheric moisture levels [15].

Hydrophobic MOFs show promise in this regard, as their resistance to chemical degradation by water [62] makes them chemically stable in humid conditions and unrestricted by competitive adsorption upon water exposure [63]. Early studies explored hydrophobic materials such as Zn-based [64] MOFs and -CF_3_ functionalized Ni_8_-based [65] MOFs, finding that they were well equipped for adsorption and retention of nerve agent and mustard gas simulants compared to hydrophilic MOFs such as HKUST-1 [16,64]. Strongly hydrophobic MOFs like zeolitic imidazolate frameworks (ZIFs) tend to prevent water adsorption into their pores altogether. Their adsorption isotherms are type III, indicating no appreciable uptake of H_2_O until near the saturation vapor pressure of water [56]. This absence of adsorbed water lessens the need for the previously discussed attributes of strong node–linker bonds or high connectivity, broadening the structural and topological criteria for effective MOF performance towards CWA degradation via oxidation. However, strongly hydrophobic MOFs are impractical for degradation via hydrolysis, where the presence of water is required for the reaction to proceed [66]. What is more, recent work by Wang et al. [5] showed that the activity of solid-phase MOFs towards CWA simulant hydrolysis can improve dramatically with increases in relative humidity, suggesting that the ability of generous numbers of external water molecules to enter MOF channels along with target species may be equally as important to hydrolytic degradation efficiency as the prevention of water–framework interactions.

In such cases where water adsorption in the MOF pores is needed or desired, competition for active sites can be combatted by functionalizing internal hydrophobicity. Most water-stable MOFs are classified as internally (or partially) hydrophobic [56], exhibiting adsorption isotherms that are type V. This style of uptake proceeds like that of the curve (c) in Figure 2, with low adsorption in the low-pressure region, followed by quick filling of the material to saturated adsorption capacity [50]. In these MOFs, water molecules can adsorb into the pores and be available to participate in target species hydrolysis without clustering around and reacting at open metal sites. Of course, depending on the pore size and connectivity of the MOF, this barrier preventing interactions of H_2_O molecules at active sites may lead to hydrogen bonding and pore filling of adsorbed water [67,68]. Such behavior can limit the ability of target species to adsorb and diffuse throughout the framework, which is yet another obstacle that interferes with CWA degradation rates. Establishing a perfect balance of stability, topology, and water affinity that diminishes these challenges would be a major stride in developing ideal MOFs for the given application. This was the focus behind our recent publication, where we investigated the behavior of environmental water at various loadings in NU-1000 [15], a Zr-MOF with combined features of strong node–linker bonds, large pore volumes, and internal hydrophobicity. Computational results demonstrated that water in NU-1000 exhibits a structural phase somewhere between liquid and vapor. This indicates that water–framework interactions are not strong enough to cause water molecules to uniformly distribute to available active sites and fully exist in the vapor phase. In contrast, water–water interactions are not strong enough to cause hydrogen-bonded clusters that fully condense into the liquid phase. These findings suggest that MOFs with the listed features may allow atmospheric water to enter and participate in target species hydrolysis without competing for adsorption on active sites or threatening structural stability, leading to potentially feasible and efficient detoxification under varying humidity conditions. Studies have also shown that MOFs without intrinsic water stability or hydrophobicity can be chemically modified to acquire these features for aqueous applications [69,70], suggesting that MOFs with only one or two desired attributes may still be worth considering.

### 3.2. Nerve Agent Hydrolysis

In the heterogeneous catalytic hydrolysis of organophosphorus-based nerve agents, MOFs with Zr_6_ nodes and displaceable -OH and -OH_2_ ligands such as UiO-66, NU-1000, and MOF-808 are among the fastest synthetic catalysts reported to date [6,7,19,71,72,73]. While the effectiveness of these MOFs towards nerve agents and simulant hydrolysis has been widely examined both experimentally and computationally, several puzzles still exist regarding the most kinetically favored hydrolysis mechanism, the performance of solid-state materials, and the role of environmental water. This section overviews the existing research findings and details areas lacking sophisticated insights.

#### 3.2.1. Proposed Hydrolysis Mechanisms in Zr-MOFs

**Scheme 2:**
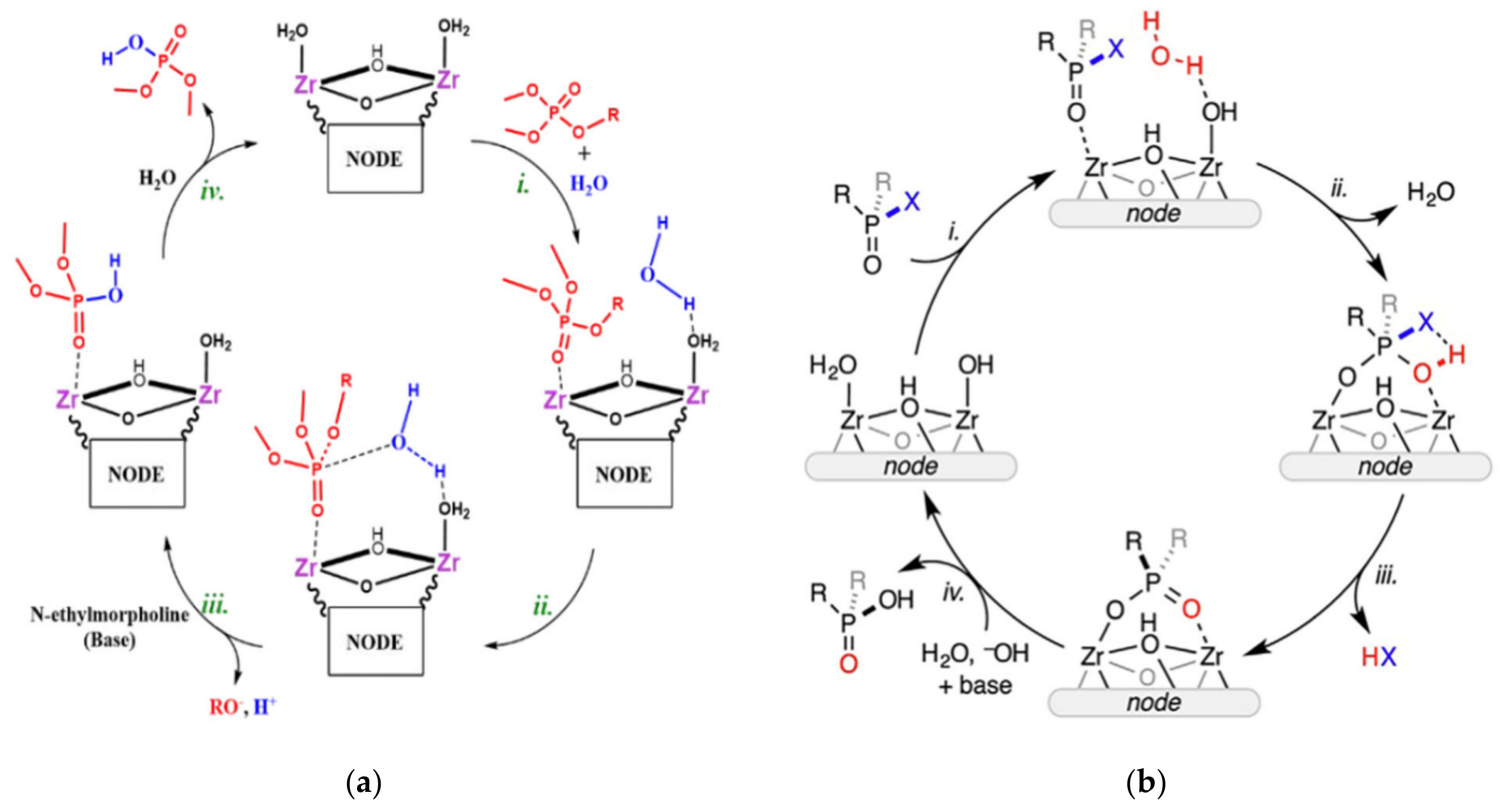
Mechanistic scheme for hydrolysis of organophosphorus nerve agents and their simulants on Zr^IV^-MOFs. (**a**) Reaction with nucleophilic attack by displaced -OH_2_ and monodentate product binding on metal nodes (example using DMNP) [75]. (**b**) Reaction with nucleophilic attack by adjacent -OH and bidentate product binding on metal nodes [73]. Reprinted with permission from ref. [75] (**left**) and ref. [73] (**right**). Copyright 2021 Royal Society of Chemistry and 2020 American Chemical Society, respectively.

Two main mechanisms are commonly proposed for the hydrolysis of organophosphorus nerve agents and their simulants in Zr-MOFs. These pathways are mainly differentiated by the participation of free H_2_O and the resulting mode of nerve agent binding on the MOF SBU (secondary building unit, i.e., inorganic metal cluster), as shown in Scheme 2. The elementary steps are as follows: (i) binding of the organophosphorus compound to an open Lewis acidic metal site; (ii) nucleophilic attack at phosphorus by either an external water molecule (Scheme 2a) or by the ligand group (OH or OH_2_) that terminates the adjacent metal site of the node (Scheme 2b); (iii) elimination of the leaving group from the organophosphorus compound by scission of the P-X bond (P-F bond in GB and GD, P-S bond in VX); and (iv) removal of the monodentate (Scheme 2a) or bidentate (Scheme 2b) hydrolyzed product from the active site [3,73,74,75].

**Figure 3:**
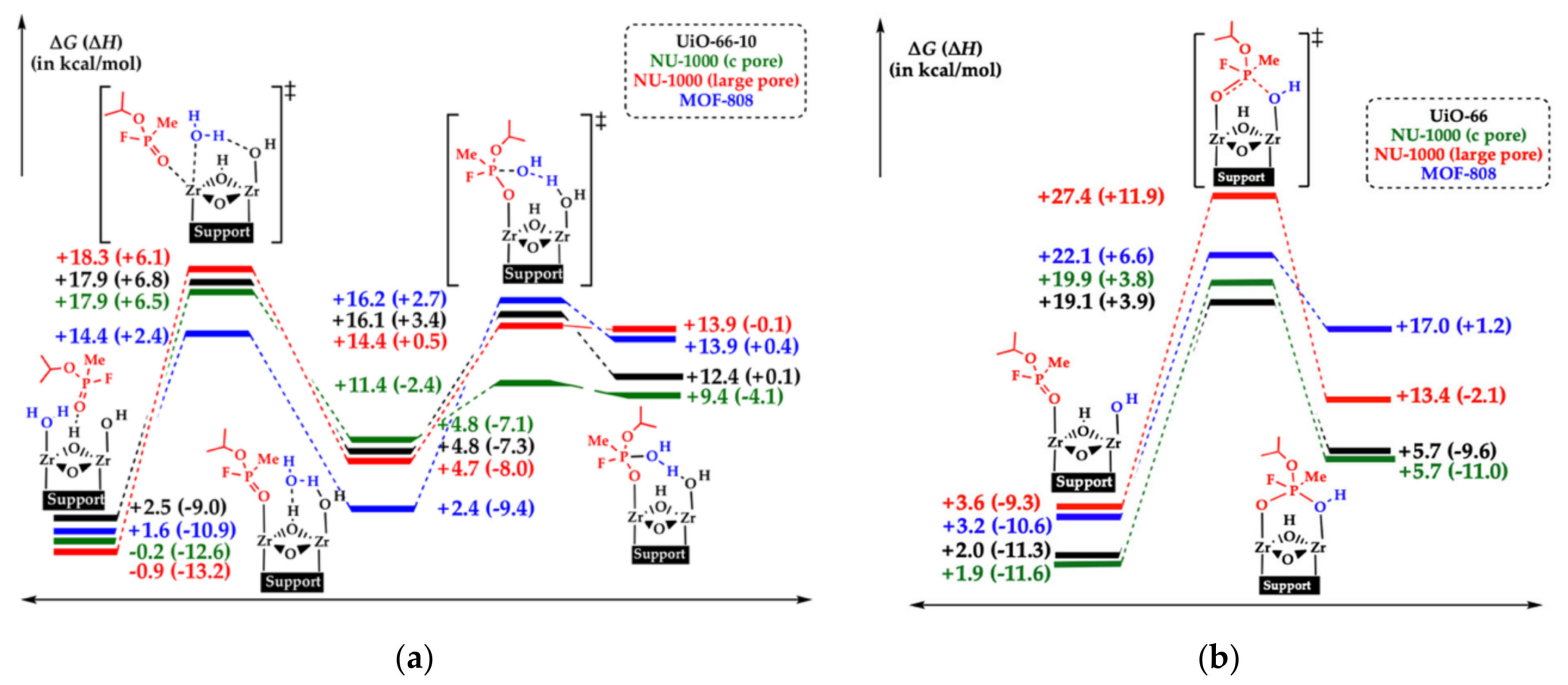
Free energies and enthalpies for hydrolysis of sarin on Zr^IV^-MOFs. (**a**) Reaction on hydrated metal nodes. (**b**) Reaction on dehydrated metal nodes [74]. Reprinted with permission from ref. [74]. Copyright 2018 American Chemical Society.

Several factors contribute to the likelihood of a given mechanism, and competing opinions exist on node configurations and reaction steps that are most kinetically favorable. The first step of -OH_2_ displacement during nerve agent coordination to the Zr-MOF seen in Scheme 2a,b is often considered rate-limiting. Evidence of this is demonstrated in a DFT study by Momeni and Cramer [74], who evaluated the energetics of sarin hydrolysis on hydrated and dehydrated Zr-MOF nodes, as shown in Figure 3. The proposed hydrated reaction pathway corresponds to steps i–iii of Scheme 2a, while the proposed dehydrated pathway resembles steps i–iii of Scheme 2b but with coordination of the nerve agent to an open metal site rather than one occupied by an -OH_2_ ligand. The results for the hydrated pathway (Figure 3a) reveal that on UiO-66 and NU-1000 nodes, the displacement of the -OH_2_ group by GB requires higher energy to reach the transition state than the nucleophilic attack of GB by the free water molecule. For this reason, preliminary dehydration of the SBUs has been widely considered to improve hydrolytic efficiency in these MOFs.

Discussions on SBU dehydration are often associated with NU-1000, an excellent Lewis acid catalyst due to the high concentration of easily accessible Lewis acidic oxozirconium clusters in its structure [76]. Dehydration of these clusters further enhances Lewis acidity, which can, in some cases, improve the activity of the MOF towards the catalytic breakdown of nerve agents. An example of this was presented in an experimental study on nerve agent removal in Zr-MOFs by Mondloch et al. [7], who observed that intentional dehydration of the nodes of NU-1000 (in an aqueous pH 10 buffered solution) accelerated its hydrolysis of the nerve agent simulant DMNP by 13.5 min. However, unlike in step iv in Scheme 2a,b, this study found that the NU-1000 node did not rehydrate to its original node configuration throughout hydrolysis. This is a potentially promising result, as it implies that the rate-limiting -OH_2_ displacement step may be continuously avoided during consecutive hydrolysis cycles in dehydrated NU-1000 (as opposed to just one initial cycle). On the other hand, one may raise a question of whether the reported efficiency of hydrolysis is simply dependent on the reaction conditions, given that a lack of participation by external water has frequently been demonstrated to introduce more problems than it solves.

As shown in Figure 3, for instance, Momeni and Cramer [74] found the activation free energies associated with the nucleophilic attack on GB by a terminal OH group in dehydrated UiO-66, NU-1000, and MOF-808 to be substantially higher than the activation free energies for the same materials using H_2_O as the nucleophile and -OH as a general base. In addition to this increased energy barrier, the absence of H_2_O in step ii of the dehydrated reaction mechanism is proposed to result in hydrolysis products binding to the SBU in a bidentate mode, corresponding to strong interaction energies that could increase the likelihood of product inhibition [3]. Support of this was found in the computational work of Troya [77], who similarly determined that the lowest-energy reaction path of GB on dehydrated UiO-66 includes the binding of the IMPA product in a bidentate manner, with a measured binding energy 80.1 kJ/mol stronger than the binding energy of monodentate IMPA from hydrolysis on hydrated UiO-66. Convincing evidence of potentially irreversible product binding was also provided in a DFT study by Mendonca et al. [3], who measured the binding energies of hydrolysis products on several Zr^IV^-MOF nodes and found that all the bidentate anions of GB, GD, and VX had strong binding with the tested SBUs of NU-1000, defective UiO-66, and MOF-808 (∆G_bind_ < −70 kJ/mol). Consequently, the reaction between nerve agents in dehydrated MOFs is thought to likely be non-catalytic under realistic environmental conditions, especially if an external water source is unavailable or uninterested in displacing the reacted phosphonate group in step iv of the hydrolysis mechanism (post-HX elimination) [74,77].

The role of external water in accelerating nerve agent removal was recently emphasized in the work of Liao et al. [19], who discovered that the operation of NU-1000 and MOF-808 in aqueous solution rather than under vacuum [57,77] resulted in a striking increase in the number of catalytic turnovers that each MOF could execute in the degradation of DMNP. This finding was attributed to the increase in the availability of water for hydrolysis, the ability of liquid water to displace some fraction of reaction-inhibiting product species from the metal node, the ability of liquid water to solubilize and stabilize displaced products, and the availability of an external water reservoir to dilute displaced products and render them less competitive as node sorbents [19]. On the basis that many potentially catalytic Zr-MOFs can recruit substantial amounts of water from humid air [19], these results lead to the conclusion that there is potential for multiple catalyst turnovers to be observed during hydrolysis in Zr-MOFs in the realistic solid-state application. Unfortunately, few papers have attempted to study the specific role of varying environmental water levels in each hydrolysis mechanism stage.

We note that current understandings of the hydrolysis mechanism largely depend on a range of assumptions, one of the most improbable being that nerve agents will have no competition with atmospheric water when attempting to adsorb at open metal sites. As a result, the level of interference external water molecules might have in the initial coordination and consistent recoordination of nerve agents to Zr-MOF nodes is not well understood. For example, a combined DFT and AIMD study by Chen et al. [78] investigated the hydrolysis of DMNP in NU-1000 and found that external H_2_O exhibits unfavorable binding with dehydrated (distorted) NU-1000 metal nodes. The calculated binding free energy of a single water molecule at an open metal site was +29 kJ/mol, suggesting that nerve agents and their simulants would not likely have to compete with water to bind to Zr sites in this MOF. This aligns with the previously discussed experimental findings of Mondloch et al. [7], which indicated that dehydrated NU-1000 did not experience rehydration throughout DMNP hydrolysis in a buffered solution. However, a more recent experimental study on GB adsorption in NU-1000 by Son et al. [66] demonstrated completely contradictory results to those of Mondloch et al. [7] and Chen et al. [78], reporting that hydrated NU-1000 outperformed dehydrated NU-1000 in the uptake of GB under both wet and dry conditions. The preliminary removal of aqua ligands was shown to have enhanced the hydrophilicity of the MOF, thus enhancing interactions between water molecules and active sites and preventing the binding of CWA molecules to the dehydrated SBUs.

The discrepancies in the literature between separate accounts of similar systems draw attention to the need for broader research efforts that capture the full picture of nerve agent hydrolysis. Many questions remain unanswered regarding the role of environmental water, along with several other reaction variables that have been virtually addressed. If water molecules have no interest in interacting with the MOF, will they be present in the vicinity of the metal nodes to participate in the reaction? If water molecules are too interested in interacting with the MOF, will nerve agents realistically be able to beat them to active sites or displace them? What about the affinity of different nerve agents for different metal site environments? What about the affinity of different nerve agents for water and vice versa? All these factors are expected to influence the role of water in the reaction mechanism and are thus expected to influence the mechanism itself. We encourage more thorough computational study from the quantum level to better understand the role of water in nerve agent hydrolysis and to begin exploring the possibility of diverse reaction mechanisms based on the type of nerve agent and the type of MOF.

#### 3.2.2. Topology and Reaction Conditions

Regarding the Zr-MOF hydrolysis energetics reported by Momeni and Cramer [74] in Figure 3a, we emphasize the differences in transition state structures between MOFs of different types (and even between different pore environments within a single MOF). Of particular interest is the case of MOF-808, whose rate-determining step was not found to be water displacement like the other materials but the nucleophilic attack by displaced H_2_O. While details of the reaction mechanism are often varied, possible support of this observation was found in a DFT study by Koning et al. [79] on the degradation of Novichok nerve agents by MOF-808, which similarly reported nucleophilic attack by an external H_2_O at the P atom of the CWA as the highest activation energy transition state of the associated hydrolysis reaction mechanism. Differences in the variations of transition state free energies from the H_2_O displacement step to the nucleophilic attack step for each system in Figure 3a indicate that the structuring of organic linkers greatly impacts the local environment and electronic structure of the metal node [74]. The topological differences between pristine UiO-66, NU-1000, and MOF-808 with respect to pore structure and node coordination are illustrated in Table 1, along with their reported surface areas, water affinities, adsorption capacities, structural stabilities, and reusabilities.

Of the three Zr-MOFs, defect-free UiO-66 has the smallest linker connectivity and pore sizes. Each Zr_6_ node of pristine UiO-66 is connected to twelve small BDC linkers, yielding pore apertures of only 6–11 Å in size. Studies of adsorption and chemical reactions in UiO-66 [56,80] show that these features limit guest species interactions to the external surface of the MOF, rendering only about 0.5% of the metal nodes catalytically active [14]. Further evidence of this effect is presented in investigations of Zr-MOF hydrolysis of DMNP by Mondloch et al. [7], who showed that the large 10–31 Å cages and low connectivity of NU-1000 (8-coordinated) enabled a much larger percentage of nodes to act as catalysts for simulant hydrolysis compared to UiO-66. The facilitated delivery of target species to the interior of the MOF resulted in a half-life for hydrolysis of DMNP that was remarkably shorter with NU-1000 (t_1/2_ = 15 min) than with defect-free UiO-66 (t_1/2_ = 45 min).

**Table 1:** Structure and properties of selected Zr-based MOFs: UiO-66, NU-1000, and MOF-808. Zr, green; O, red; C, gray; and hydrogen atoms are omitted for clarity [9,14]. Node and structure illustrations reproduced with permission from refs. [9,14]. Copyright 2015 Wiley-VCH.

Properties	Zr-Based Metal–Organic Frameworks		
	UiO-66	NU-1000	MOF-808
SBU	[Zr_6_(*u_3_*-O)_4_ (*u_3_*-OH)_4_]^12+^	[Zr_6_(*u_3_*-O)_4_ (*u_3_*OH)_4_(H_2_O)_4_ (OH)_4_]^8+^	[Zr_6_(*u_3_*-O)_4_ (*u_3_*-OH)_4_(HCOO)_6_]^6+^
Organic linker	BDC^2−^	TBAPy^4−^	BTC^3−^
Connectivity	12-connected ditopic	8-connected tetratopic	6-connected tritopic
Topology	fcu	csq	spn
Window size (Å)	6	31	14
Pore size (Å)	8/11	10/31	4.8/18.4
Node	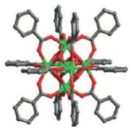	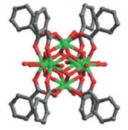	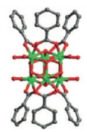
Structure	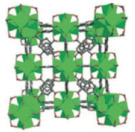	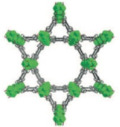	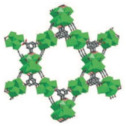
Surface area (m^2^/g)	~1200 [44]	~2300 [81]	~2000 [82]
Water affinity	Hydrophilic	Internally hydrophobic	Hydrophilic
Water adsorption capacity (g/g)	~0.4 [83]	~1.0 [84]	~0.6 [83]
Structural stability *	Stable in water and acid/base conditions [85]	Stable in water and acid/base conditions [86]	Stable in water and acid/base conditions [87]
Reusability	Reusable, robust cycling performance in water [83]	Reusable, subject to capillary-force-driven channel collapse with repeated cycles in the presence of water [88]	Reusable, significant decrease in surface area with repeated cycles in the presence of water [83]

BDC^2−^ = benzene-1,4′-dicarboxylate (terephthalate); BPDC^2−^ = biphenyl-4,4′-dicarboxylate; BTBA^4−^ = 4,4′,4″,4‴-(biphenyl-3,3′,5,5′-tetrayltetrakis(ethyne-2,1-diyl))tetrabenzoate; TBAPy^4−^ = 1,3,6,8-tetrakis(*p*-benzoate)pyrene; BTC^3−^ = benzene-1,3,5-tricarboxylate. * Stability in the range of pH = 0–11, instability in the form of ligand leaching reported for all three Zr-MOFs at pH = 12 and higher [86].

That said, a later study by Moon et al. [14] showed that despite NU-1000 having the largest pore sizes of the three MOFs, the 6-connected MOF-808 (5–18 Å pores) was found to have by far the shortest hydrolysis rate of DMNP at t_1/2_ = 0.5 min. Key geometrical and energetic data measured in the DFT study by Momeni and Cramer [74] showed an increasing trend in Zr-H_2_O bond distances and a decreasing trend in Mayer bond order and electrophilicity indices of these sites in each MOF with decreasing linker coordination, suggesting that the binding of water to Zr atoms is weaker in MOFs with lower linker connectivity. This would explain the higher energetic favorability towards H_2_O displacement in hydrated MOF-808 than in hydrated UiO-66 or NU-1000 and the tendency of MOF-808 to have the highest kinetic efficiency of the three materials. It was also found in the work of Mendonca et al. [3] that the binding free energies of water molecules at Zr-MOF nodes weaken in the order of NU-1000 (c pore) > defective UiO-66 > NU-1000 (large pore) > MOF-808, indicating that pore size plays a supplementary role in the water–node interaction strength. Such results suggest that the most crucial topological design rule for efficient nerve agent removal in chemically stable MOFs is the coordination of the metal nodes to the organic linkers, given that the MOFs in question are equipped with sufficiently large pores. In an MOF like NU-1000 (whose metal sites are accessible from two very different pore environments), the preferential pore location of nerve agent molecules should therefore be considered, as this preference could potentially dictate the ability of the nerve agent to displace -OH_2_ when attempting to bind to the metal node.

**Figure 4:**
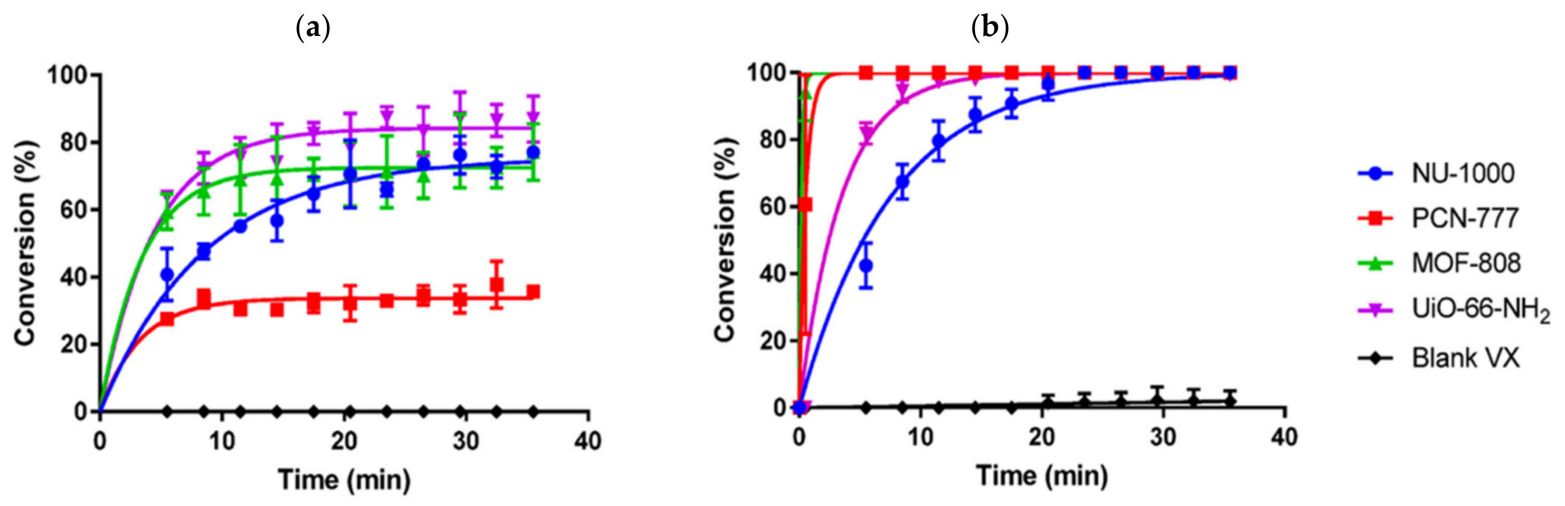
Degradation rates of VX in various aqueous-phase Zr-MOFs. (**a**) Pure water solution. (**b**) NEM buffer solution [90]. Reprinted with permission from ref. [90]. Copyright 2017 American Chemical Society.

Until now, most of the CWA decontamination kinetics reported in the literature have not been for MOFs in the solid phase but rather in solution. Furthermore, in many of the discussed findings, the Zr-MOFs are not utilized in neat water but in aqueous media with specific pH values achieved by adding buffers [89]. The effectiveness of these materials for degradation has, therefore, largely depended on the presence of a buffer solution, which facilitates the reaction, deprotonates water molecules, and removes unwanted acidic hydrolysis byproducts [6]. Evidence of this was explicitly demonstrated in the work of de Koning et al. [90], who measured the degradation rates of VX in the presence of several Zr-MOFs in solutions of N-ethylmorpholine (NEM) buffer vs. pure water. As shown in Figure 4, all the tested MOF catalysts achieved greatly enhanced hydrolysis of VX when operating in a pH 10 buffer solution.

When executed in water, organophosphate hydrolysis was slower or even absent and often incomplete due to catalyst poisoning from nerve agent degradation products occupying catalytic sites [90]. We also emphasize that the trends in conversion % versus time with respect to each type of MOF do not carry over from Figure 4a,b, as is evident from PCN-777 having the least efficient degradation of VX in pure water while having the most efficient degradation of VX (along with MOF-808) in NEM buffer. These results suggest that current research efforts employing hydrolysis by MOFs in buffered solution may not provide an adequate evaluation of the features of promising MOFs for their realistic application as heterogeneous catalysts in protective equipment. Reports of hydrolysis by aqueous-phase MOFs (even in pure water) also fail to provide insight into the crucial effects of atmospheric moisture levels.

**Figure 5:**
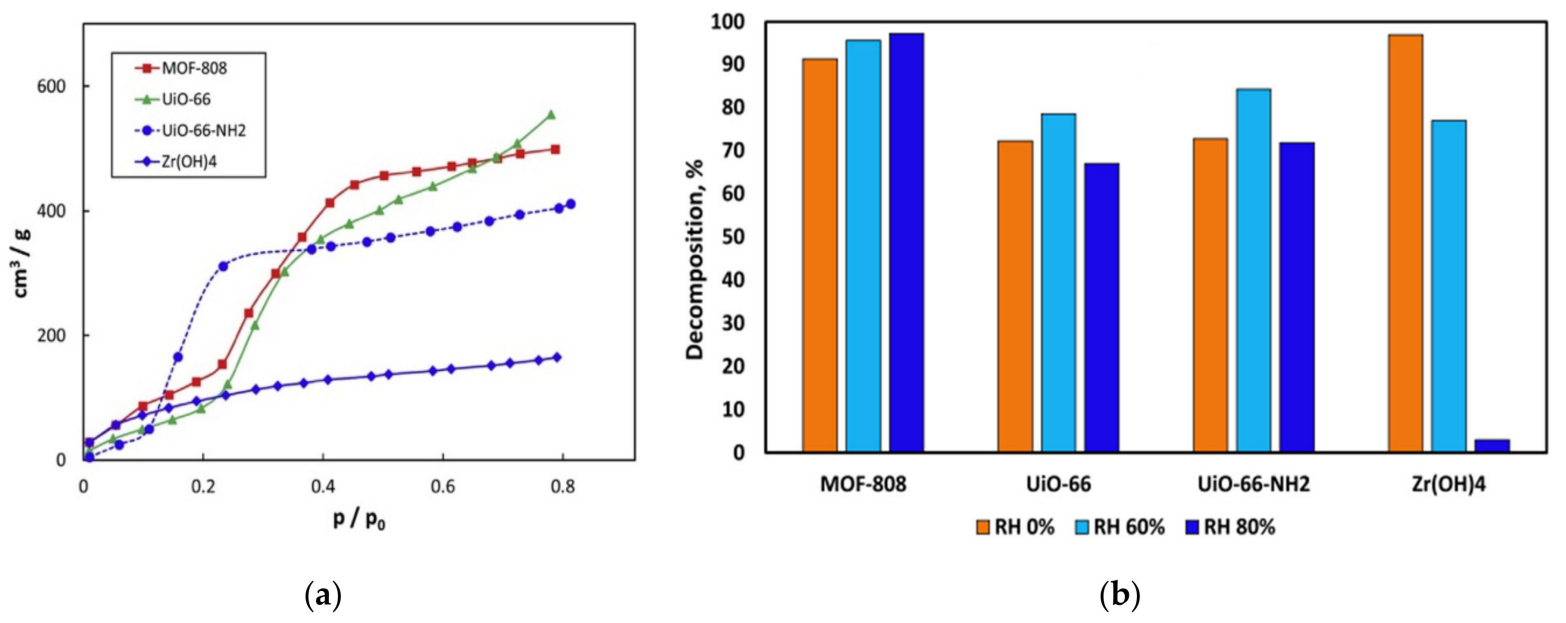
Properties of Zr-based reactive materials exposed to water in the solid phase. (**a**) Water adsorption isotherms at room temperature; (**b**) comparison of GD degradation performances at t = 5 min according to pretreatment conditions of 0, 60, and 80% RH [71]. Reprinted with permission from ref. [71]. Copyright 2019 Elsevier.

One of the few works considering the solid-phase implementation of MOFs under humid conditions is that of Ryu et al. [71], who evaluated the impact of water loading on Zr-MOF functionality towards hydrolysis of GD and VX. By measuring degradation rates under pretreatment conditions of 0, 60, and 80% RH, it was found that UiO-66, the amino-functionalized UiO-66-NH_2_, and MOF-808 all showed a high decomposition ability of both nerve agents regardless of the air humidity conditions. Comparison of these Zr-MOFs to the relatively more hydrophilic Zr-based catalyst Zr(OH)_4_ provided additional insight into the impact of water affinity on hydrolytic performance, as shown in Figure 5.

Water adsorption isotherms in Figure 5a demonstrate that UiO-66, MOF-808, and UiO-66-NH_2_ exhibit type II or type IV adsorption, while Zr(OH)_4_ exhibits type I adsorption [71]. This indicates that Zr(OH)_4_ has a high degree of hydrophilicity, which was shown to reduce the ability of the material to decompose CWAs in humid environments. The study reported that Zr(OH)_4_ was capable of efficient nerve agent decomposition but only before the active sites on the nodes became blocked by water molecules at ~80% RH, as shown in Figure 5b. Interestingly, while UiO-66 and MOF-808 are indeed more hydrophobic than Zr(OH)_4_, these MOFs’ adsorption isotherms indicate unrestricted water adsorption. Water condensation in the pores of both materials occurs from 20 to 40% relative humidity, which is considered unusually low for water-stable MOFs [56]. Such results imply that an internally hydrophobic Zr-MOF like NU-1000 may exhibit even more efficient hydrolysis than these materials under humid conditions.

Regarding topology, the findings of Ryu et al. [71] followed the trends observed for the same MOFs in buffer solution, showing that the degradation rates of nerve agents by MOF-808 were greater than those by the UiO-66 series. As seen in Figure 5b, the hydrolytic efficiency achieved by MOF-808 was impressive, with >90% decomposition of GD in under 5 min. This result does not necessarily align with expectations, considering that solid-phase decomposition lacks a high pH buffer to accelerate hydrolysis and neutralize phosphate acid products that bind to and poison the MOF catalyst [5]. Investigating further, a similar analysis of the effects of water exposure on CWA decontamination kinetics was found in the work of Wang et al. [5], who explored GD, VX, and DMNP hydrolysis in solid-phase UiO-66, UiO-66-NH_2_, and NU-1000. This study had vastly different results than those of Ryu et al. [71], showing overall slower hydrolysis rates and very different reactivity trends in solid-state decontamination than in solution decontamination. Comparisons of GD hydrolysis rates of Zr-MOFs in buffer solution [90,91] to those in the solid phase from Ryu et al. [71] and Wang et al. [5] are given in Table 2.

The comparisons in Table 2 reveal the extent to which the works by Ryu et al. [71] and Wang et al. [5] contradict one another. Ryu et al. [71] observed >80% degradation of GD by UiO-66 and UiO-66-NH_2_ in under 10 min, while Wang et al. [5] did not observe >80% hydrolysis for either MOF until ~24 h. Moreover, Wang et al. [5] observed that GD hydrolysis rates for the UiO-66 series were much faster than GD hydrolysis rates for NU-1000, which has larger pore sizes, lower connectivity, and is more hydrophobic. Differing trends were also observed with respect to changes in humidity level. Results by Ryu et al. [71] suggested that the impact of increasing humidity on the initial hydrolysis rates of GD in all three Zr-MOFs was mild. In the parallel analysis conducted by Wang et al. [5] using DMNP, the results showed that increases in the content of environmental water led to moderate increases in the hydrolysis rates of NU-1000 and UiO-66-NH_2_ but significant increases in the hydrolysis rate of UiO-66.

Conversion time aside, both studies observed that the hydrolysis rates of solid-phase UiO-66 and UiO-66-NH_2_ (at all humidity levels) were comparable, which is not typical for these MOFs in a buffered solution. Functionalizing organic linkers in MOFs has frequently been explored for reducing reaction barriers and increasing catalytic activity [74,92,93]. In the case of UiO-66, amino functionalization of the linkers has been shown to vastly enhance nerve agent hydrolysis rates in the presence of a buffer [14] due to the ability of the amino moieties to act as proximal bases, transferring protons at crucial portions of the catalytic cycle [93,94]. When the MOF is instead added to water without a buffer, the weak acidity of the aqueous solution makes it difficult for amine groups to exhibit proton transfer abilities as Brønsted bases [71]. This conclusion is computationally supported in the work of Islamoglu et al. [95], whose DFT calculations of DMNP hydrolysis in aqueous UiO-66-NH_2_ revealed that deprotonation of nearby water in step iii of the hydrated reaction mechanism was more energetically favored when promoted by -OH bound to Zr than when promoted by a proximal amino group. This would explain why the amino functionalization of UiO-66 in an aqueous solution of neat water or the solid phase appears to have little influence on its kinetic efficiency towards nerve agent hydrolysis. Combined with the results in Table 2, these findings further prove that the design rules that enhance the hydrolysis rates of Zr-MOFs in aqueous solution do not necessarily apply to solid-phase decontamination [5]. However, the degree to which they may differ is unclear, given the scattered nature of the few available inquiries into the solid-phase application.

We note that research efforts have begun shifting towards addressing the dependency of efficient hydrolysis on aqueous-phase catalysts in the presence of a buffer, and current discoveries appear to be promising. In a report by Moon et al. [6], the polyethyleneimine (PEI) polymer was investigated as a heterogenous buffer for nerve agent and simulant hydrolysis in aqueous NU-1000. The results showed that dehydrated NU-1000 could hydrolyze DMNP, GD, and VX with half-lives of 1.8 min, 3.8 min, and 12.7 min, respectively, indicating a strong potential for efficient removal in heterogeneous systems. More recently, publications have advanced to investigating the feasibility of incorporating basic species into MOFs in the solid phase. An example of this was presented in the work of Ma et al. [96], who discovered that combining Zr-MOFs with crosslinked PEI-based hydrogel supplied the Lewis acidic sites, catalyst-regenerating base, and plentiful water needed for boosting near-instantaneous hydrolysis of GD and VX under ambient conditions. The use of composites was also studied by Luo et al. [97], who showed that incorporating imidazole into the pores of MOF-808 formed a material that structurally mimicked the phosphotriesterase (PTE) enzyme from soil bacteria (which is highly efficient in catalyzing the hydrolysis of organophosphorus compounds in nature), making it capable of rapid DMNP hydrolysis under high humidity conditions. Continued research efforts in this direction are encouraged, as they are necessary for further developing MOFs that can function as intended in personal protective equipment. We point out, though, that selecting appropriate materials for future studies will likely be contingent on establishing distinct structure–property relationships for MOFs operating in the solid phase.

### 3.3. Sulfur Mustard Oxidation

**Scheme 3:**
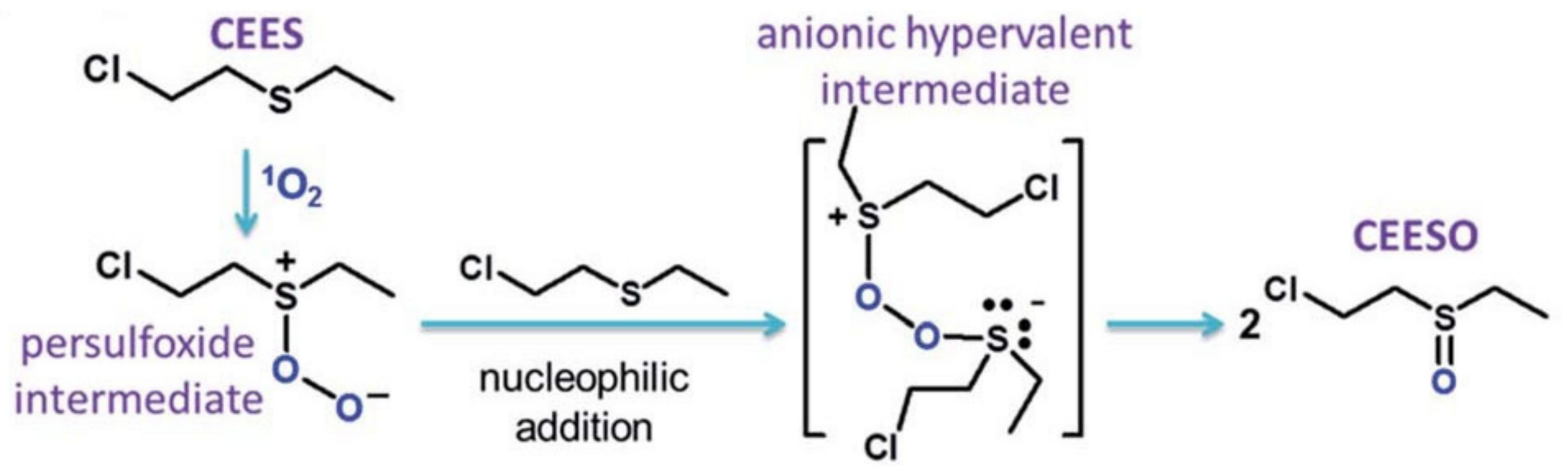
Proposed mechanism for the oxidation of CEES by singlet oxygen (^1^O_2_) [35]. Reprinted with permission from ref. [35]. Copyright 2016 Royal Chemical Society.

As stated previously, oxidation is thought to be the most effective strategy for the degradation of sulfur mustard, and selective partial oxidation is necessary for achieving decomposition into a nontoxic product. Complete selective oxidation requires a mild oxidizing agent, as strong agents such as hydrogen peroxide or *tert*-butyl hydroperoxide are often observed to generate both partially and fully oxidized products [35,98]. The most desirable mild oxidant is singlet oxygen (^1^O_2_), a reactive species commonly produced from ground state O_2_ using a photosensitizer [99]. Unfortunately, many prominent photosensitizers have a proclivity to aggregate in aqueous media, which diminishes their ability to absorb light and produce ^1^O_2_ [35,98,100]. MOFs are an attractive potential solution to this problem, as their tunable networks allow easy incorporation and post-synthetic modification of an array of discrete photoactive moieties at their organic linkers, and their 3D structures allow those moieties to be isolated by surrounding metal nodes [11,101]. Studies of MOFs for the removal of HD and its simulant CEES are thus primarily centered around ^1^O_2_ photooxidation, the proposed mechanism of which is presented in Scheme 3.

Like nerve agent hydrolysis, the most promising and most frequently investigated MOFs for HD photooxidation are Zr-based. In addition to chemical stability, the high valence metal nodes in Zr-MOFs offer excellent thermal stability and reusability [101], making them especially appealing supports for generating singlet oxygen [102] and subsequent catalytic and selective oxidation [99]. Many research efforts have thus been motivated to design and utilize Zr-MOFs as photocatalysts. Meaningful findings and advancements in this field are addressed in the following subsections.

#### 3.3.1. Photooxidation in Zr-MOFs

**Figure 6:**
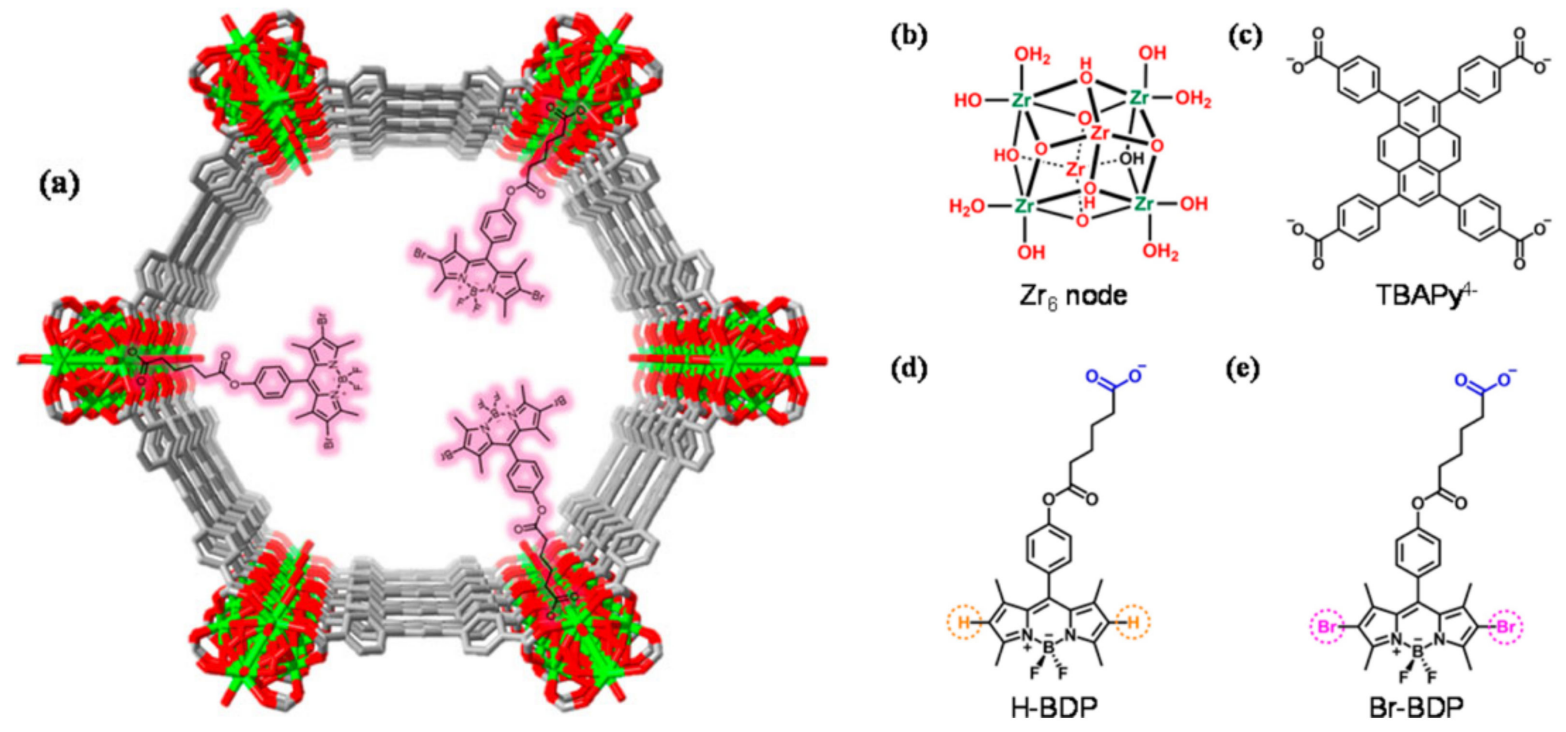
(**a**) Schematic representation of Br-BDP@NU-1000; (**b**) Zr_6_ node of NU-1000, with SALI-displaceable aqua and hydroxy ligands shown in green; (**c**) structure of organic linker of NU-1000, (**d**) H- BDP, and (**e**) Br-BDP [99]. Reprinted with permission from ref. [99]. Copyright 2017 American Chemical Society.

Early reports on the impressive potential of Zr-MOFs for the photooxidation of HD can be found in a collection of papers by Liu et al., which demonstrated that the pyrene-containing NU-1000 [35] (TBAPy linkers) and the porphyrin-containing Zr-based PCN-222/MOF-545 [98] (TCPP linkers) were able to selectively oxidize the HD simulant CEES to 2-chloroethyl ethyl sulfoxide (CEESO) with half-lives of 6 and 13 min, respectively. Following these findings, research extended to a more detailed photooxidation strategy involving the photosensitizers in Zr-MOFs as nonstructural ligands [99,103]. A study by Atilgan et al. [99] examined this strategy through post-synthetic modification of NU-1000 via solvent-assisted ligand incorporation (SALI), in which aqua and hydroxo groups on Zr_6_ nodes were displaced by boron-dipyrromethene (BODIPY) photoactive moieties. A schematic representation of the modified structure can be seen in Figure 6. Experimental evaluations of the catalytically synthesized Br-BDP@NU-1000 shown in Figure 6a revealed that BODIPY-incorporated NU-1000 yielded a CEES photooxidation half-life of ~2.5 min, which is more than twice as efficient as that of unaltered NU-1000. While this result suggests that nonstructural ligands are far more photoactive than structural organic linkers, we note that similar photooxidation performance has been observed in MOFs whose structural linkers were strategically synthesized to have photoactive components.

As shown in a later work by Zhang et al. [104], when the porous and robust UiO-68 Zr-MOF was de novo functionalized with photoactive triazolobenzothiadiazole (TBTD)-conjugated terphenyldicarboxylate (TPDC) linkers, it could efficiently and selectively photocatalyze CEES into CEESO with a half-life as low as 3 min (nearly as fast as the post-synthetically BODIPY-modified NU-1000). Goswami et al. [105] similarly demonstrated that a version of the PNC-57 Zr-MOF partially substituted with benzoselenadiazole linkers (PCN-57-Se) was able to catalyze the photooxidation of CEES to CEESO with 100% conversion within 12 min (t_1/2_ = 3.5 min). It was, however, shown in comparison that PNC-57 partially substituted with benzothiadiazole linkers (PNC-57-S) took 25 min (t_1/2_ = 7.5 min) to selectively oxidize CEES, indicating that slight changes in the chemical structure of photosensitizers can also have a significant influence on photooxidation efficiency. As another example of this, when determining which of the two BODIPY derivatives in Figure 6e,d (H-BCP and Br-BDP) to incorporate into NU-1000, Atilgan et al. [99] found that homogeneous H-BDP engendered much slower conversion of CEES than homogeneous Br-BDP despite both species possessing the same chromophore. Understanding what properties contribute to higher or lower activity levels in photosensitizers is critical to selecting practical photoactive species for initial or post-synthetic functionalization into Zr-MOFs. In the case presented by Goswami et al. [105], time-resolved emission spectroscopy and supporting DFT calculations pointed to efficient excited-state singlet-to-triplet intersystem crossing for selenium-containing samples as a key factor in the higher catalytic activity of PCN-57-Se compared to PCN-57-S, amongst other potential contributors. We urge the increased utilization of these time-dependent DFT approaches, as they could be a highly beneficial tool for identifying ideal MOF–photosensitizer pairs.

As an alternative to complex DFT calculations, the ^1^O_2_ generation quantum yield (Φ_Δ_) of a photosensitizer is a quantitative measure of its photoactive performance that can provide useful insight into its potential for promoting efficient HD photooxidation. This was demonstrated in the work of Buru et al. [102], who used singlet oxygen quantum yields of photoactive species in Zr-MOF linkers to hypothesize that UMCM-313 (perylene linkers; Φ_Δ_ = 0.45) would have a shorter half-life for partial oxidation of CEES than the previously studied PCN-222/MOF-545 [98] (porphyrin linkers; Φ_Δ_ = 0.38) and NU-1000 [35] (pyrene linkers; Φ_Δ_ = 0.40). Experiments revealed that the rates of CEES oxidation by each MOF were indeed positively correlated to the ^1^O_2_ quantum yield of each MOF’s linkers, where PCN-222/MOF-545 (t_1/2_ = 11 min) < NU-1000 (t_1/2_ = 6 min) < UMCM-313 (t_1/2_ = 4 min) [102]. Nevertheless, certain photoactive species have operational requirements that, regardless of their ^1^O_2_ generation abilities, critically hinder their practical application. For example, C_60_-fullerene is known to be a strong singlet oxygen generator (Φ_Δ_ = 1) [106], and Howarth et al. [103] demonstrated that post-synthetic incorporation of fullerene-based photosensitizers into NU-1000 resulted in the photooxidation of CEES with a half-life of only 3.5 min (under UV-LEDs). However, the low solubility of fullerenes makes them a challenge to synthetically produce, and their low absorption efficiency across the visible spectrum limits their ability to be studied further for practical applications [99,106]. Porphyrins are also known to have excellent ^1^O_2_ generating capabilities and are a prime focus in combination with MOF structures due to their ease of synthesis and exceptional chemical stability. However, they are similarly hindered by their strong absorption in the UV region and weak absorption in the visible region [107].

**Figure 7:**
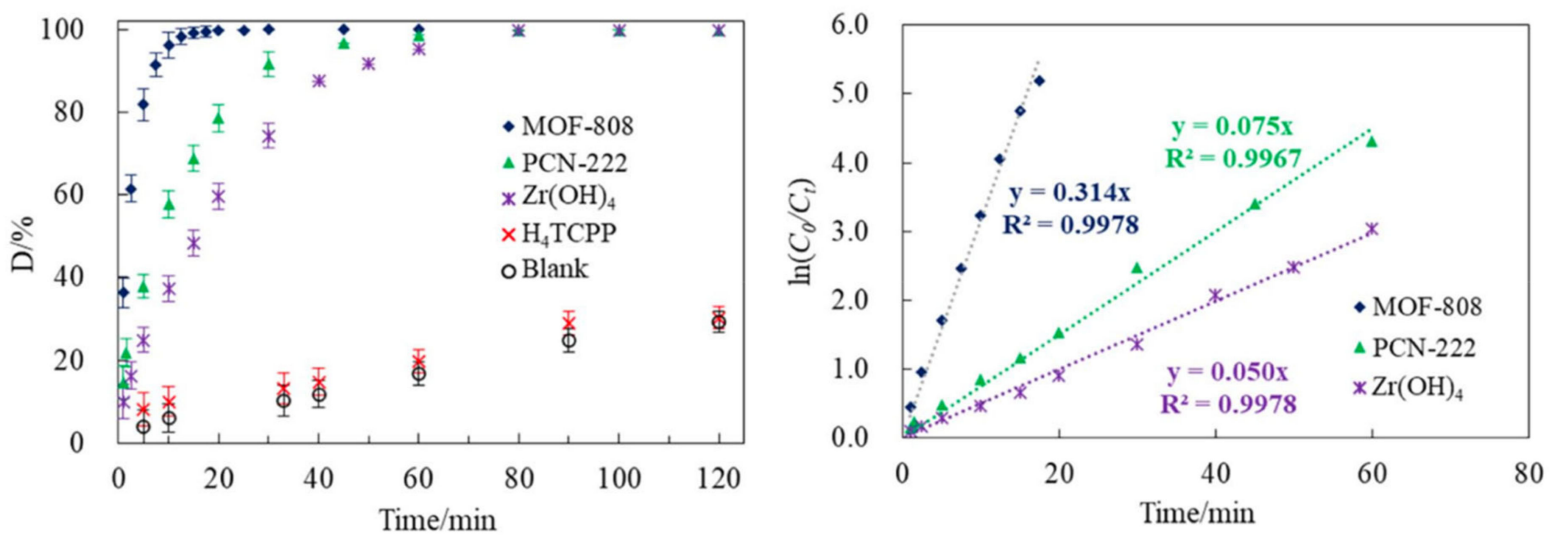
Kinetic profiles for catalytic oxidation of HD in H_2_O_2_ ethanol solution using different materials as catalysts under standard room lighting [108].

This drawback was displayed in the work of Wu et al. [108], who investigated HD oxidation by H_2_O_2_ in different Zr-based materials and found that degradation of HD in blank reaction solution under standard room lighting did not accelerate with the addition of pure H_2_TCPP, the porphyrin ligand of the PCN-222 Zr-MOF. As shown in Figure 7, the reaction solution containing PCN-222 did achieve more enhanced catalytic activity towards HD degradation under standard room lighting than the blank solution, but this was attributed to the catalytic Zr_6_ sites and three-dimensional framework contributed by the MOF rather than the presence of porphyrinic linkers [108]. Such findings help draw attention to the fact that the reported performances of many of the photosensitizers discussed in this section rely on protic solvents and/or consistent exposure to light of specific wavelengths. While it is clear from these reports that photosensitizers can be successfully functionalized into MOFs in various ways, their intended purpose may ultimately be a challenge to realistically exercise.

Although essential strides in the realm of HD photooxidation by MOFs have been made with respect to understanding different photosensitizers and methods of their implementation, most efforts have failed to provide any correlation between degradation efficiency and Zr-MOF topology. Systematic research on this issue was recently kickstarted by Hao et al. [101], who compared the efficiency and selectivity of three Zr-porphyrin MOFs with different pore shapes, sizes, and metal node connectivity for the photooxidation of CEES. The results showed that CEES reaction rates were correlated to the surface area of the MOFs, where larger surface areas and pore volumes could accommodate more reaction substrate (CEES) and enhance the diffusion of substrates and products, leading to faster oxidation [101]. This aligns with an earlier report on the diffusion of 2-CEES through NU-1000 and UiO-66, which stated that the larger pore sizes of NU-1000 facilitated better rates and activation energies of simulant transport [10]. We also see from Figure 7 that in the investigations of HD oxidation by Wu et al. [108], PCN-222 and MOF-808 were found to be more efficient for H_2_O_2_ activation and HD degradation than Zr(OH)_4_, which was attributed to the larger surface areas of the MOFs enhancing HD adsorption and facilitating HD interactions with H_2_O_2_ coordinated to the Zr_6_ nodes. Oddly enough, however, the results also show that MOF-808, with a measured S_BET_ of 1344 m^2^/g and a pore volume of 0.82 cm^2^/g, exhibited better catalytic performance for HD oxidation than PCN-222, which had a reported specific surface area of 1625 m^2^/g and a pore volume of 1.22 cm^2^/g [108]. This suggests that the topological aspects contributing to oxidation efficiency in these materials are more complicated than surface area and pore volume alone. More thorough investigations will undoubtedly be needed to establish design rules for HD photooxidation in Zr-MOFs and determine if those rules correspond to design rules that facilitate nerve agent hydrolysis.

#### 3.3.2. Tuning Enhanced Photocatalytic Activity

**Figure 8:**
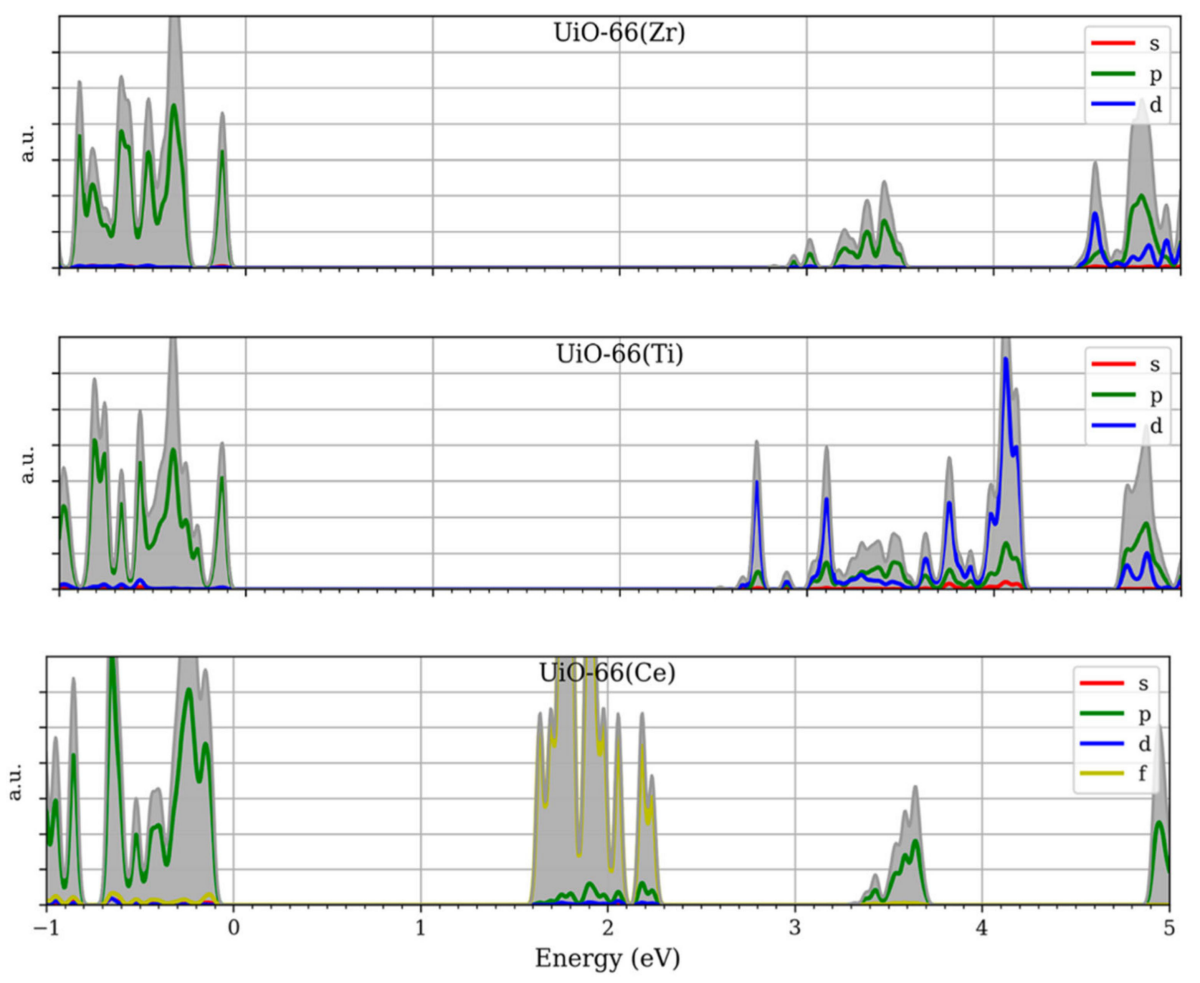
Total (gray) and projected (red, green, blue, and yellow) density of states of UiO-66(Zr), UiO-66(Ti), and UiO-66(Ce). The energy is expressed with respect to the Fermi energy (E_F_ = 0) [109]. Reprinted with permission from ref. [109]. Copyright 2018 American Chemical Society.

Photocatalysis in MOFs ideally occurs through the excitation of an electron from a photoactive ligand or linker, which transfers to a nearby metal node to create a redox active center [109]. In recent years, research efforts on the use of MOFs as photocatalysts have begun exploring structural tuning in the form of linker functionalization or metal node substitutions, as these types of modifications can increase the ability of an MOF to absorb light for excitation or to transfer excited electrons from ligands to metal sites [110,111]. Evidence of this was first demonstrated by Gomes Silva et al. [112], who investigated the effects of amino-functionalization on the light absorption properties of UiO-66. While Zr-based UiO-66 is structurally well suited for photocatalysis, its practical application is hindered by its large HOCO-LUCO band gap, which impedes excitation with light in the visible region of the spectrum [109]. Gomes Silva et al. [112] found that adding NH_2_ groups to the UiO-66 framework causes a shift in its adsorption spectrum towards the visible range, improving its ability to operate as a photocatalyst. The reasoning behind this was later detailed in the work of Hendrickx et al. [113], who showed that functional groups incorporated into UiO-66 yield orbital contributions that reduce the effective band gap of the MOF, easing the excitation of linker electrons. Following this discovery, Hendrickx et al. [109] began exploring how changes to the UiO-66 metal nodes would affect its electronic properties. As elucidated in Figure 8, LMCT (ligand-to-metal charge transfer) in pristine UiO-66 is hindered by the lack of overlap between its empty zirconium d states and empty linker band [109]. The DOS plots indicate that substituting titanium into the metal nodes of UiO-66 introduces 3d states that are sufficiently low in energy to change the original LUCO from ligand- to metal-based while simultaneously overlapping the linker orbitals [109]. Combined experimental and TD-DFT methods confirmed that even doping with titanium gives rise to node-localized excitation peaks in the UiO-66 excitation spectrum, resulting in improved charge transfer abilities and increased photocatalytic activity.

With all this in mind, a similar study by Wu et al. [110] suggested that the electronic structure of MOFs can be specifically engineered for desired reactions using thoughtful combinations of metal node substitution and linker functionalization. DFT calculations demonstrated that UiO-66 substituted with Ce^4+^, for example, has strong potential as a photocatalyst due to its favor towards LMCT. Support of this can be found in Figure 8, which shows the appearance of a broad band of empty 4f orbitals in UiO-66(Ce) that significantly lowers the LUCO state of the material, leading to a negative E_LMCT_ [109,110]. However, too negative E_LMCT_ values can prevent visible light adsorption in MOFs [110], limiting their ability to act as photocatalysts in applications that require excitation from natural light. Subsequent evaluations by Wu et al. [110] revealed that linker functionalization of UiO-66(Ce) with electron-withdrawing groups is a procedure that can successfully raise LMCT energy while preserving favorable charge separation capability, with increasing numbers of functional groups on each linker enhancing this effect (as long as E_LMCT_ remains negative). While not centered around CWA degradation specifically, these findings suggest that combined linker functionalization and metal substitution in MOFs is a promising research avenue for enhancing photoactive performance under realistic environmental conditions and thus for accelerating the practical implementation of MOF/photosensitizer materials as catalysts for HD photooxidation.

## 4. Summary and Outlook

This review summarizes experimental and computational studies on the use of MOFs for nerve agent hydrolysis and sulfur mustard selective oxidation. Owing to their unprecedented structural stability, zirconium-based MOFs are commonly considered the most promising materials for both degradation mechanisms. Zr-MOFs with additional features of wide channels, low linker coordination, and internal hydrophobicity have been shown to present as especially strong candidates for hydrolysis applications, given the large volume available for guest species adsorption and diffusion, the enhanced accessibility of the metal nodes, and the ability of environmental water to adsorb in the pores and participate in hydrolysis without competing for active sites. Water adsorption isotherms are a popular method for characterizing overall water affinity, providing insight into whether water will adsorb in and interact with a given MOF. However, they do not offer much insight into local water interactions at the linkers, at the nodes, and in different pore environments. Developing computational approaches to identify and assign these local regions of hydrophilicity and hydrophobicity could be a major step forward in designing MOFs with the perfect balance of water affinity for efficient hydrolysis.

While high-valence Zr metal nodes are ideal for creating strong and stable bonds with carboxylate organic linkers, they also tend to create strong bonds with guest molecules and nonstructural ligands. For this reason, displacement of aqua ligands and strongly bound reaction products present as rate-limiting hydrolysis steps in many Zr-MOFs. Combined experimental and computational studies have uncovered that certain structural and topological attributes can significantly reduce the influence of these variables on hydrolytic efficiency. There is a positive correlation between linker coordination and Zr-H_2_O bond strength, where the binding of water ligands to Zr atoms is weaker in MOFs with lower connectivity. Guest–host bond strengths and transition state structures have also been shown to vary in MOFs with different pore sizes and even in different pore regions within the same MOF. Future studies would benefit from considering nerve agent location when evaluating hydrolysis rates in MOFs, especially those with sites accessible from multiple pore environments.

In terms of structural tuning, linker functionalization and SBU dehydration have frequently been presented as promising strategies for improving hydrolysis kinetics. Unfortunately, these design rules stem primarily from implementing MOFs in aqueous buffer solutions, which facilitate the reaction and do not account for environmental variables relevant to the solid-phase application. Studies have shown that functionalizing proximal bases only reduces reaction barriers and enhances catalytic activity in a high pH environment. Convincing arguments have also been made that displaced water in the vicinity of the metal nodes is crucial in the solid state for preventing bidentate product binding and promoting product displacement, both of which are necessary to avoid catalyst inhibition. Evidence suggests that this effect would be accelerated with the addition of external water molecules from the atmosphere; however, well-developed understandings of the role of environmental water (at different loadings) in the hydrolysis mechanism are lacking. The proposed mechanisms appear to carry the assumption that all nerve agents and Zr-MOFs have an equal affinity for water, often failing to incorporate the participation of external water altogether. These mechanisms also neglect to consider the level of interaction between a nerve agent and MOF, suggesting that the degradation of all nerve agents in all Zr-MOFs will proceed by the same reaction steps. More thorough ab initio calculations are undeniably needed to accurately characterize the role of environmental water at critical steps of the hydrolysis process and to begin exploring the possibility of diverse reaction mechanisms based on the type of nerve agent and the type of MOF.

In addition to their structural stability, the thermal stability and reusability of Zr-MOFs make them stand out as appealing supports and catalysts for sulfur mustard degradation via ^1^O_2_ photooxidation. While many Zr-MOFs contain photoactive species in the form of structural organic linkers, studies have shown that enhanced photocatalytic activity can sometimes be achieved by post-synthetic incorporation of photoactive species in the form of nonstructural ligands. Whether or not the method of incorporation improves photooxidation efficiency depends on several factors, including the chemical structure of the photosensitizer and the MOF–photosensitizer pair. Evaluating singlet oxygen generation quantum yields of photoactive species is a useful first step to take when determining which MOF to use (or which photosensitizer to incorporate into a given MOF), as this property has proven to be a reasonable indicator of the partial oxidation efficiency a material will likely achieve. However, determining truly ideal MOF–photosensitizer pairs requires a more in-depth evaluation of chemical properties from the quantum scale. Time-dependent density functional theory (TD-DFT) is an evolving technique that can shed light on these properties, alleviating the troublesome task of hypothesizing favorable materials and comparing their performances experimentally. Advancements in TD-DFT approaches are expected to be instrumental in elucidating the key factors that determine whether a certain photosensitizer or incorporation method will improve an MOF’s photocatalytic capabilities. Improved characterizations of structure–property relationships are also needed, as MOFs’ structural and topological design rules governing effective sulfur mustard removal are not currently well understood.

Insights into optimizing photooxidation in Zr-MOFs are far less developed than those for optimizing hydrolysis, and significant efforts in many areas will be needed to advance with the practical application. Current studies reporting efficient MOF performance are almost exclusively from operation in protic solvents and under LED lights, used to improve selectivity and enhance light absorption and excitation, respectively. Many high-functioning photosensitizers realistically exhibit weak absorption in the visible spectrum region, severely challenging their practical use under standard room lighting or in the dark. Investigations on MOFs for alternative photocatalysis applications have exhibited a breakthrough in this area, revealing that linker functionalization and metal node substitution are methods of structural tuning that can enhance photocatalytic activity without any external stimuli. Such modifications have been shown to alter the electronic properties of Zr-MOFs, increasing their ability to absorb light for excitation and/or transferring excited electrons from ligands to metal sites. Further development in this area could vastly reduce the barriers preventing photosensitizer excitation in relevant field conditions, essential to the realistic employment of Zr-MOFs for sulfur mustard photooxidation.

Several notable approaches to improving the practical implementation of Zr-MOFs for CWA degradation have emerged in recent years. An obvious question concerning solid-state nerve agent removal is the feasibility of hydrolysis in dry environments, given that water is necessary for the reaction to proceed and for the removal of poisonous byproducts. Integrating MOFs with catalyst-regenerating bases is a promising technique for mimicking the role of high pH buffers in the solid phase, and the development of MOF composites incorporating these features is currently underway [96,97]. Composites have also been explored in sulfur mustard oxidation applications, naturally to integrate the catalytic abilities of Zr-MOFs with the photoactive abilities of materials that exhibit strong absorption in the visible range [114]. Alternatively, studies have also investigated MOF composites containing known oxidants to explore the oxidation of sulfur mustard by methods other than ^1^O_2_ photooxidation [115]. Advancements in atomistic understandings of mechanisms are a challenge for hydrolysis and oxidation reactions, given that the locations and positions of active metal sites in MOFs are difficult to identify and control. Single-atom catalysts have arisen as a useful strategy in this regard, as they offer a platform for enhanced catalytic performance where the identification of reaction processes on a molecular level can be more easily realized [116,117,118]. We note that the proven ability of Zr-MOFs to efficiently perform both nerve agent hydrolysis and sulfur mustard oxidation has made them of principal focus in growing inquiries surrounding dual-function MOF catalysts [8,119,120]. That said, the emerging research efforts discussed in this paragraph are not exclusive to zirconium-based MOFs, and recent studies on dual-function degradation suggest that Zr-MOFs are not the only materials worth considering [121].

## Figures and Tables

**Table 2:** GD hydrolysis rates found in the literature for UiO-66, UiO-66-NH_2_, MOF-808, and NU-1000. The time to reach 80% conversion is shown for each MOF in pH 10 buffer solution versus each MOF in the solid phase at ambient humidity.

MOF	Time to Reach 80% Conversion of GD		
	pH 10 Buffer Solution	Ryu et al. [71]	Wang et al. [5]
UiO-66	~10 min [91]	5–10 min	~1 day
UiO-66-NH_2_	~4 min [91]	5–10 min	~1 day
MOF-808	<1 min [90]	<5 min	-
NU-1000	<1 min [90]	-	>5 days

## Data Availability

No new data were created or analyzed in this study. Data sharing is not applicable to this article.
